# μ-Chlorido-bis­{[1-benzyl-3-(2,4,6-tri­methyl­phen­yl)imidazol-2-yl­idene-κ*C*]silver(I)} chloride 1,2-di­chloro­ethane hemisolvate

**DOI:** 10.1107/S2414314624008617

**Published:** 2024-09-10

**Authors:** Kotiba Malek, Kuppuswamy Arumugam

**Affiliations:** a Department of Chemistry, Wright State University, 3640 Colonel Glenn Hwy, Dayton, OH 45435, USA; Purdue University, USA

**Keywords:** crystal structure, N-heterocyclic carbene, silver chloride, bis­(NHC) silver complex

## Abstract

The title compound, [Ag_2_(C_19_H_20_N_2_)_4_]Cl·0.5C_2_H_4_Cl_2_, can be readily generated by treatment of (1-benzyl-3-(2,4,6-tri­methyl­phen­yl)imidazolium chloride with sodium bis­(tri­methyl­sil­yl)amide followed by silver chloride. The mol­ecular structure of the compound was confirmed using NMR spectroscopy and single-crystal X-ray diffraction analysis. The crystal structure of the title compound at 110 K has monoclinic (*P*2_1_/*c*) symmetry. The represented silver compound is of inter­est with respect to anti­bacterial properties and the structure displays a series of weak inter­molecular hydrogen-bonding inter­actions with the chloride counter-anion.

## Abstract 2

The solid-state structural analysis of the title compound reveals that the two mol­ecules of bis­(1-benzyl-3-mesitylimidazol-2-yl­idene)silver are connected *via* a bridging chloride atom. The structure also reveals non-classical inter­molecular hydrogen-bonding inter­actions involving the chloride counter-anion.

## Graphical abstract

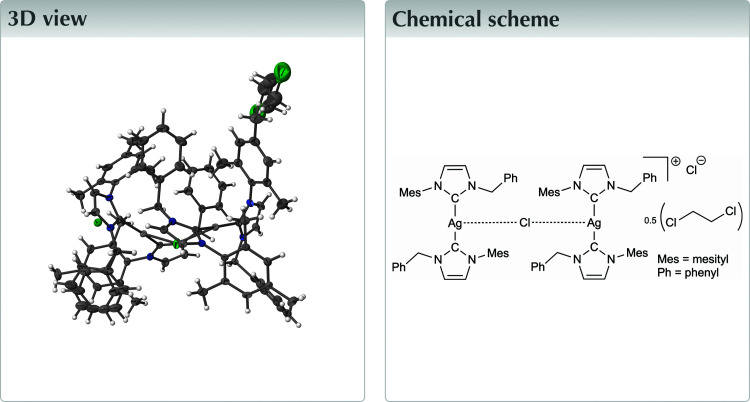

## Structure description

Recent research has focused on discovering new and more effective silver-based anti­bacterial compounds. In 2004, Young’s group reported a variety of silver(I) complexes containing N-heterocyclic carbenes as a new class of anti­biotics (Melaiye *et al.*, 2004 ▸). N-heterocyclic carbenes (NHC) form strong *M*—C_carbene_ bonds (Arduengo *et al.*, 1991 ▸) that are far more stable than most phosphines due to their increased σ-donation, as well as π-back-donation from metal to carbene (Jafarpour *et al.*, 1999 ▸; Herrmann & Köcher, 1997 ▸). The stability and versatility of these ligands allow them to serve as metal carriers for transition metals such as copper, gold, and silver in biological media (Medici *et al.*, 2016 ▸). Subsequently, NHC-containing silver complexes have been targeted for the slow release of silver ions under biological conditions (Streciwilk *et al.*, 2014 ▸; Karatas *et al.*, 2016 ▸; Aher *et al.*, 2014 ▸; A Patil *et al.*, 2020 ▸). In relevance to this context, we prepared the title compound and studied its solid and solution-state structural features. The leading results pertaining to the title compound are presented below.

**Figure 1:**
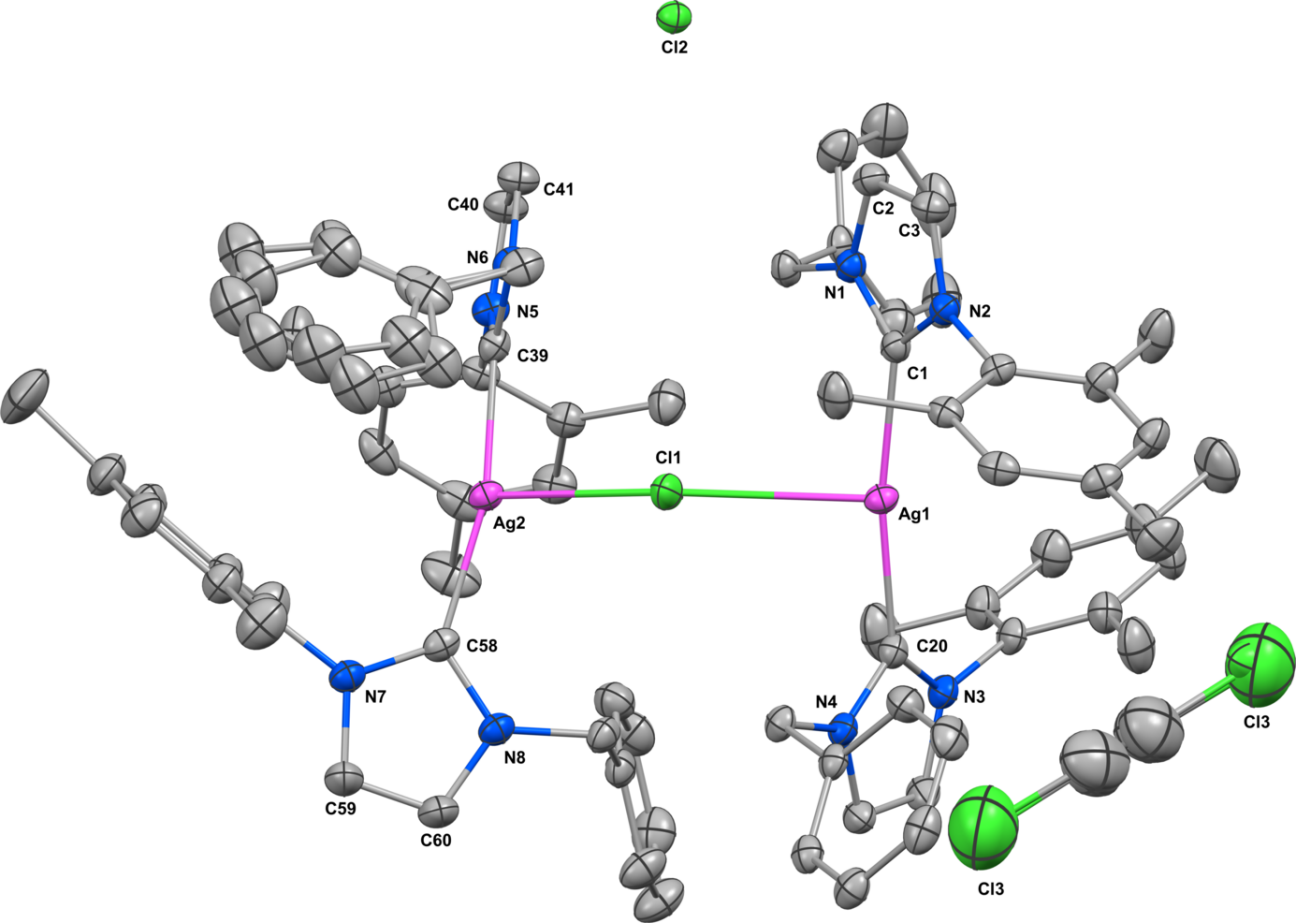
The mol­ecular structure of the title compound with solvate, the displacement ellipsoids drawn at the 50% probability level. Hydrogen atoms are omitted for clarity.

**Figure 2:**
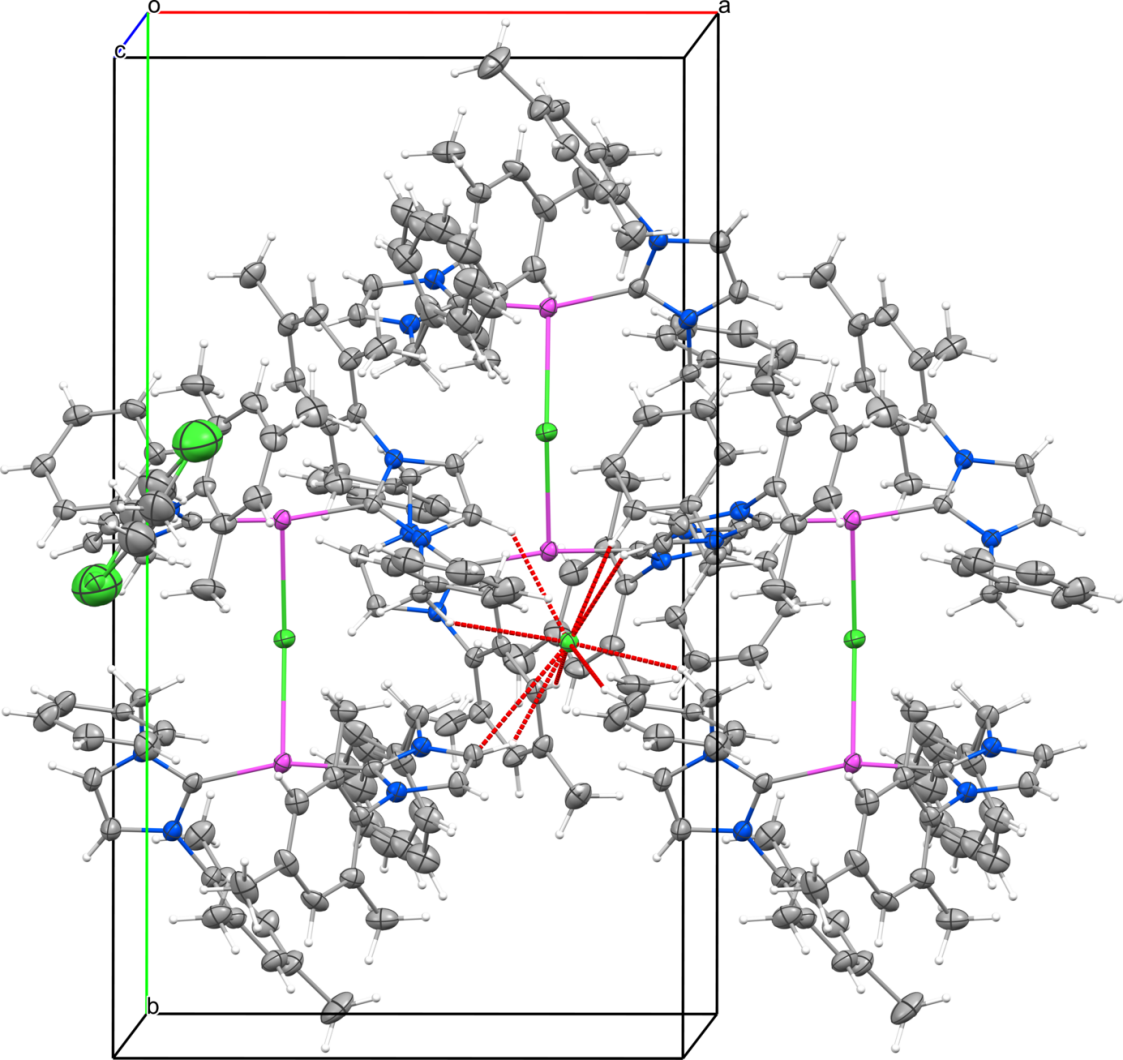
Inter­molecular C—H⋯·Cl inter­actions (dotted lines) in the title compound. Displacement ellipsoids are drawn at the 50% probability level.

The title compound crystallizes in the monoclinic space group *P*2_1_/*c* with four silver-carbene complex mol­ecules and two 1,2-di­chloro­ethane mol­ecules in the unit cell. The mol­ecular structure of the compound is presented in Fig. 1 ▸. The mol­ecular geometry around the silver atom is linear, where two NHCs are attached to the silver atom with C20—Ag1—C1 and C39—Ag2—C58 bond angles of 170.55 (8) and 163.97 (8)°, respectively. However, a chloride anion bridges the two bis­(NHC) silver units with an Ag2—Cl1—Ag1 bond angle of 148.90 (2)°. The observed Ag1—Cl1 and Ag2—Cl1 bond lengths are 2.8755 (6) Å and 2.8149 (6) Å. Subsequently, a T-shaped coordination environment is observed around the silver atom. The Ag—C_carbene_ bond lengths Ag1—C1, Ag1—C20, Ag2—C39, Ag1—C58 are 2.099 (2), 2.098 (2), 2.098 (2) and 2.104 (2) Å. These parameters are well within the reported bond parameters for Ag—C_carbene_ and C_carbene_—Ag—C_carbene_. The compound also engages in weak inter­molecular C—H⋯Cl inter­actions. A pictorial representation of the non-classical hydrogen bonding and the bond parameters are presented in Fig. 2 ▸ and Table 1 ▸, respectively.

The 1,2-di­chloro­ethane solvate mol­ecule is located on a crystallographic inversion center, and is disordered over two pseudo-mirror related moieties.

A CSD structure search for bis­[(1-benzyl-3-(2,4,6-tri­methyl­phen­yl)imidazol-2-yl­idene)silver(I)] revealed no hits. However, a few mono-NHC and bis-NHC silver complexes bearing 1-benzyl-3-(2,4,6-tri­methyl­phen­yl)imidazol-2-yl­idene or similar NHCs have been reported. A few of the bis-NHC silver complexes include, bis-[1-benzyl-3-(4-methyl­phen­yl)imidazol-2-yl­idene]silver(I) hexa­fluorido­phosphate (Huang & Qin, 2011 ▸), and mono-NHC silver complexes include di-μ-acetato-bis-[3-benzyl-1-(2,4,6-tri­methyl­phen­yl)imidazol-2-yl­idene]silver(I)] (Jayaraman *et al.*, 2019 ▸), (1-benzyl-3-mesitylimidazol-2-yl­idene)chloro­silver(i) (Samantaray *et al.*, 2011 ▸) and bis­(μ^2^-bromo)­bis­(1-benzyl-3-mesityl-2,3-di­hydro-1*H*-imida­zol-2-yl­idene)disilver (Ortiz *et al.*, 2016 ▸). In scanning the literature, the C_NHC_—Ag, C—N and N—C—N bond lengths and C_NHC_—Ag—C_NHC_ and N—C_NHC_—N bond angles are comparable to those of the title compound.

## Synthesis and crystallization

All synthetic procedures were executed under a nitro­gen atmosphere glove box (Inert Glove Box System). All glasswares were subjected to heat at 110°C for 12 h before use. The starting material 1-(benz­yl)-3-(2,4,6-tri­methyl­phen­yl)imidazolium chloride was prepared according to literature pro­cedures (Maishal *et al.*, 2009 ▸). Solvents (CH_2_Cl_2_, Et_2_O, THF, and toluene) were dried with a solvent purification system (Inert Innovative Technology, Inc.), degassed using three consecutive freeze–pump–thaw cycles and stored over 4 Å mol­ecular sieves in the glove box. The NMR solvents: CDCl_3_ (99.9%) was purchased from Acros Laboratories, dried over 4 Å mol­ecular sieves and stored in the glove box prior to use. All other chemicals were purchased commercially and used as received. The ^1^H and ^13^C NMR spectra were recorded on a Bruker 300 MHz spectrometer. Spectra were referenced to the resid­ual solvent as an inter­nal standard, for ^1^H NMR: CDCl_3_, 7.26 p.p.m. and ^13^C NMR: CDCl_3_, 77.16 p.p.m.

**Figure 3:**
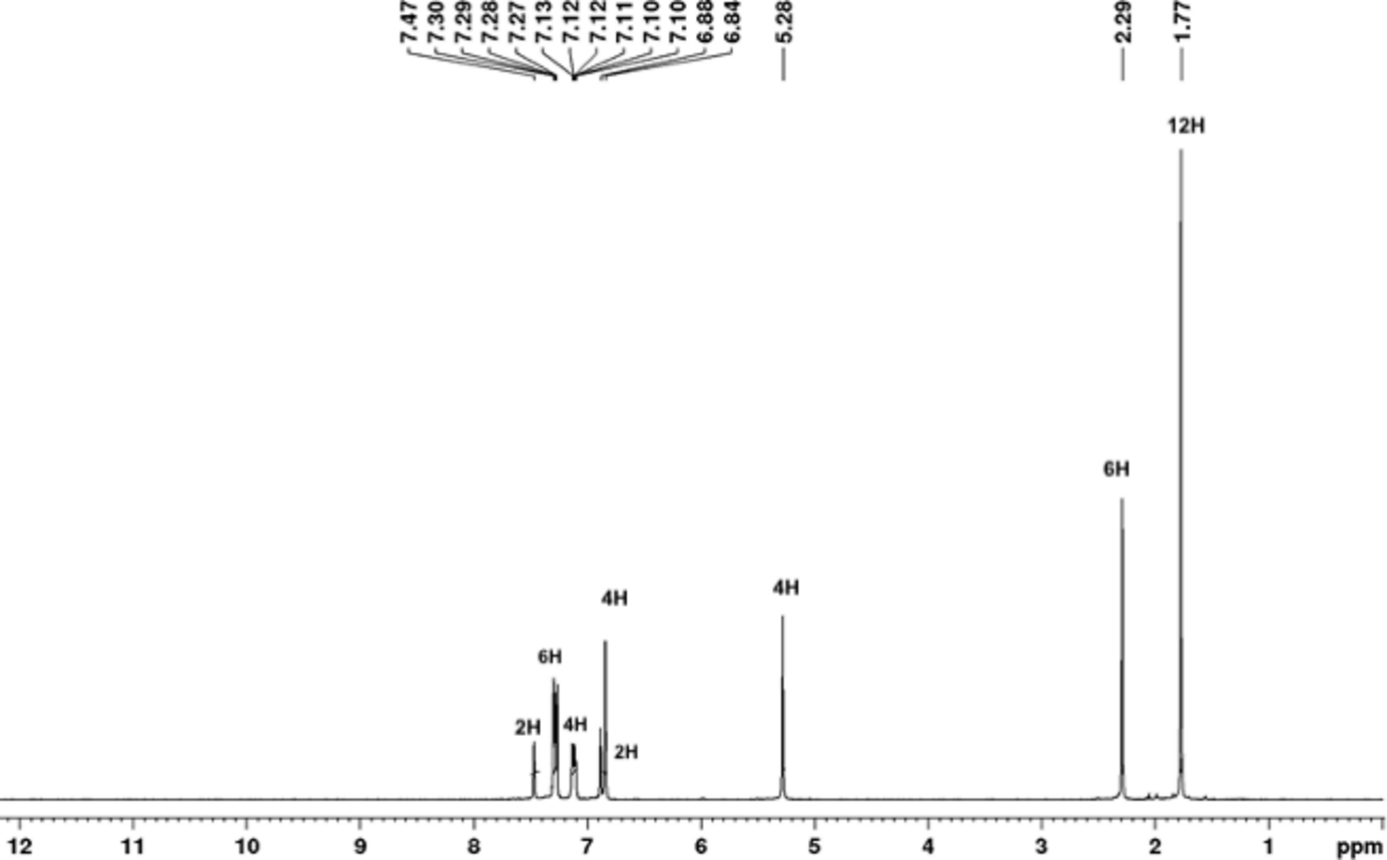
^1^H NMR of the title compound in CDCl_3_

**Figure 4:**
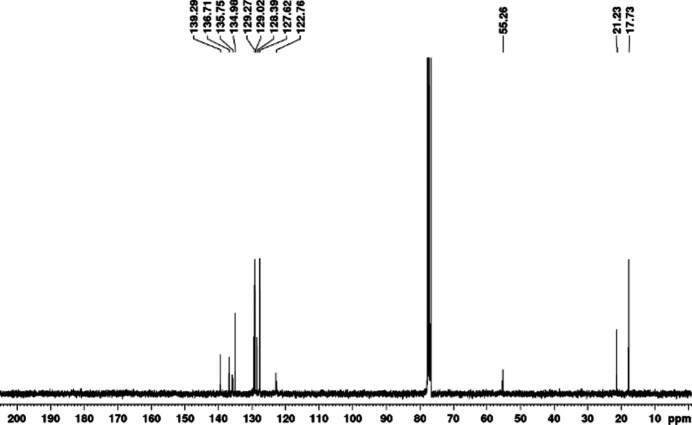
^13^C NMR of the title compound in CDCl_3_

In a 10 ml vial equipped with a stir bar, 1-(benz­yl)-3-(2,4,6-tri­methyl­phen­yl) imidazolium chloride (0.120 g, 0.384 mmol) and sodium bis­(tri­methyl­sil­yl)amide (0.077 g, 0.422 mmol) were mixed in 2 ml of toluene. After 2 h, the yellow solution was filtered through a plug of celite into a vial containing AgCl (0.0247 g, 0.173 mmol) in 2 ml of toluene. The mixture was stirred for 24 h. The resulting solution was filtered through a plug of celite and dried under vacuum. The brown residue was dissolved in minimum amount (∼2 ml) of CH_2_Cl_2_ and the product was precipitated with 15 ml Et_2_O and further washed with 3 ×10 ml Et_2_O to produce a white solid. Yield: 0.75 g, 78%. ^1^H NMR spectroscopic analysis of the silver complex proved consistent with the mol­ecular structure. The absence of the hydrogen atoms attached to the C_carbene_ in the ^1^H NMR of complexes proves the formation of a silver–carbene bond. The proton NMR (CDCl_3_) spectrum shows mesityl H atoms (*ortho*-CH_3_ and *para*-CH_3_) at 1.77 and 2.29 p.p.m., respectively. The benzylic CH_2_ H atoms were observed at 5.28 p.p.m. The 6.88 and 7.47 p.p.m. signals correspond to the C2 and C3 imidazole H atoms. The two aromatic mesityl H atoms are represented by a singlet at 6.84 p.p.m.; the rest of the H atoms corresponding to the phenyl rings were observed between 7.30 p.p.m. and 7.12 p.p.m.. All signals corresponding to the carbon atoms were observed by ^13^C NMR spectroscopy. ^1^H NMR (δ, CDCl_3_, 300 MHz): δ 7.47 (*m*, 2H), 7.30–7.27 (*m*, 6H), 7.13–7.12 (*m*, 4H), 6.88 (*s*, 2H), 6.84 (*s*, 4H), 5.28 (*s*, 4H), 2.29 (*s*, 6H), 1.77 (*s*, 12H) (Fig. 3 ▸).^13^C NMR (δ, CDCl_3_, 75 MHz): δ 139.29, 136.71, 135.75, 134.98, 129.27, 129.02, 128.39, 127.62, 122.76, 55.26, 21.23, 17.73 (Fig. 4 ▸).

Colorless crystals of the title compound were obtained by diffusing diethyl ether into a saturated solution of 1,2-di­chloro­ethane solution.

## Refinement

Crystal data, data collection and structure refinement details are summarized in Table 2 ▸. The title compound co-crystallizes with half a solvent mol­ecule of 1,2-di­chloro­methane per asymmetric unit. The chlorine and carbon atoms of the solvent mol­ecule are disordered (Cl3 and Cl3*B*; C77 and C77*B*). The positions of Cl3 and Cl3*B* as well as C77 and C77*B* are split into two. The C—Cl bond lengths of the solvate mol­ecule were restrained to a target value of 1.77 (2) Å. The C52–C57 phenyl ring was refined as disordered. The geometry (bond lengths and angles) of the two disordered moieties were restrained to be similar to those of another better defined phenyl ring (C14–C19) using SAME and SADI restraints (with an e.s.d. of 0.02 Å). For all disordered atoms *U^ij^* components of ADPs closer to each other than 2.0 Å were restrained to be similar (with an e.s.d. of 0.01 Å^2^). Subject to these conditions, the solvate disorder refined to an occupancy ratio of 0.423 (16):0.577 (16), and that of the phenyl group to 0.446 (13) to 0.554 (13).

## Supplementary Material

Crystal structure: contains datablock(s) I. DOI: 10.1107/S2414314624008617/zl4077sup1.cif

Structure factors: contains datablock(s) I. DOI: 10.1107/S2414314624008617/zl4077Isup2.hkl

CCDC reference: 2380960

Additional supporting information:  crystallographic information; 3D view; checkCIF report

## Figures and Tables

**Table 1:** Hydrogen-bond geometry (Å, °)

*D*—H⋯*A*	*D*—H	H⋯*A*	*D*⋯*A*	*D*—H⋯*A*
C2—H2⋯Cl2	0.95	2.80	3.573 (2)	140 (1)
C8—H8⋯Cl2	0.95	2.81	3.669 (2)	157 (1)
C13—H13*A*⋯Cl2	0.99	2.91	3.784 (2)	147 (1)
C21—H21⋯Cl2	0.95	2.78	3.567 (2)	141 (1)
C38—H38⋯Cl2	0.95	2.90	3.779 (2)	155 (1)
C41—H41⋯Cl2	0.95	2.66	3.419 (2)	137 (1)
C70—H70*A*⋯Cl2	0.99	2.74	3.634 (2)	151 (1)

**Table 2:** Experimental details

Crystal data	
Chemical formula	[Ag_2_(C_19_H_20_N_2_)_4_]Cl·0.5C_2_H_4_Cl_2_
*M*_r_	1441.59
Crystal system, space group	Monoclinic, *P*2_1_/*c*
Temperature (K)	110
*a*, *b*, *c* (Å)	12.7605 (2), 22.4142 (4), 25.0233 (4)
β (°)	93.055 (1)
*V* (Å^3^)	7146.9 (2)
*Z*	4
Radiation type	Cu *K*α
μ (mm^−1^)	5.79
Crystal size (mm)	0.25 × 0.21 × 0.2

Data collection	
Diffractometer	Xcalibur, Sapphire3
Absorption correction	Analytical (*CrysAlis PRO*; Rigaku OD, 2015 ▸)
*T*_min_, *T*_max_	0.545, 0.746
No. of measured, independent and observed [*I* > 2σ(*I*)] reflections	54034, 14039, 13203
*R*_int_	0.038
(sin θ/λ)_max_ (Å^−1^)	0.618

Refinement	
*R*[*F*^2^ > 2σ(*F*^2^)], *wR*(*F*^2^), *S*	0.037, 0.093, 1.06
No. of reflections	14039
No. of parameters	897
No. of restraints	281
H-atom treatment	H-atom parameters constrained
Δρ_max_, Δρ_min_ (e Å^−3^)	2.12, −0.65

Computer programs: *CrysAlis PRO* (Rigaku OD, 2015 ▸), *SHELXS* (Sheldrick, 2008 ▸), *SHELXL2019/1* (Sheldrick, 2015 ▸), *Mercury* (Macrae *et al.*, 2020 ▸) and *OLEX2* (Dolomanov *et al.*, 2009 ▸).

## full crystallographic data

### µ-Chlorido-bis{[1-benzyl-3-(2,4,6-trimethylphenyl)imidazol-2-ylideneκC]silver(I)} chloride 1,2-dichloroethane hemisolvate Crystal data

**Table:**

[Ag_2_(C_19_H_20_N_2_)_4_]Cl·0.5C_2_H_4_Cl_2_	*F*(000) = 2980
*M**_r_* = 1441.59	*D*_x_ = 1.340 Mg m^−^^3^
Monoclinic, *P*2_1_/*c*	Cu *K*α radiation, λ = 1.54178 Å
*a* = 12.7605 (2) Å	Cell parameters from 9034 reflections
*b* = 22.4142 (4) Å	θ = 2.5–3.1°
*c* = 25.0233 (4) Å	µ = 5.79 mm^−^^1^
β = 93.055 (1)°	*T* = 110 K
*V* = 7146.9 (2) Å^3^	Block, colorless
*Z* = 4	0.25 × 0.21 × 0.2 mm

### µ-Chlorido-bis{[1-benzyl-3-(2,4,6-trimethylphenyl)imidazol-2-ylideneκC]silver(I)} chloride 1,2-dichloroethane hemisolvate Data collection

**Table:**

Xcalibur, Sapphire3 diffractometer	13203 reflections with *I* > 2σ(*I*)
ω scans	*R*_int_ = 0.038
Absorption correction: analytical (CrysAlisPro; Rigaku OD, 2015)	θ_max_ = 72.2°, θ_min_ = 3.5°
*T*_min_ = 0.545, *T*_max_ = 0.746	*h* = −11→15
54034 measured reflections	*k* = −24→27
14039 independent reflections	*l* = −30→30

### µ-Chlorido-bis{[1-benzyl-3-(2,4,6-trimethylphenyl)imidazol-2-ylideneκC]silver(I)} chloride 1,2-dichloroethane hemisolvate Refinement

**Table:**

Refinement on *F*^2^	Primary atom site location: structure-invariant direct methods
Least-squares matrix: full	Secondary atom site location: difference Fourier map
*R*[*F*^2^ > 2σ(*F*^2^)] = 0.037	Hydrogen site location: inferred from neighbouring sites
*wR*(*F*^2^) = 0.093	H-atom parameters constrained
*S* = 1.06	*w* = 1/[σ^2^(*F*_o_^2^) + (0.0555*P*)^2^ + 3.6026*P*] where *P* = (*F*_o_^2^ + 2*F*_c_^2^)/3
14039 reflections	(Δ/σ)_max_ = 0.002
897 parameters	Δρ_max_ = 2.12 e Å^−^^3^
281 restraints	Δρ_min_ = −0.65 e Å^−^^3^

### µ-Chlorido-bis{[1-benzyl-3-(2,4,6-trimethylphenyl)imidazol-2-ylideneκC]silver(I)} chloride 1,2-dichloroethane hemisolvate Special details

**Table:**

Geometry. All esds (except the esd in the dihedral angle between two l.s. planes) are estimated using the full covariance matrix. The cell esds are taken into account individually in the estimation of esds in distances, angles and torsion angles; correlations between esds in cell parameters are only used when they are defined by crystal symmetry. An approximate (isotropic) treatment of cell esds is used for estimating esds involving l.s. planes.
Refinement. Aromatic (C—H) H atoms were added using a riding-model approximation with C—H bond lengths of 0.95 Å with *U*_iso_ (H) = 1.2 *U*_eq_(C_ar_H). Methyl (CH_3_) H atoms were treated as a rotating group and added using a riding-model approximation to the carbon atom to which they are attached. Methyl H atoms were fixed at a distance of 0.98 Å with *U*_iso_ (H) = 1.5 *U*_eq_(CH_3_). Methylene (CH_2_) H atoms were added using riding-model approximations with a C—H bond distance of 0.99 Å.

### µ-Chlorido-bis{[1-benzyl-3-(2,4,6-trimethylphenyl)imidazol-2-ylideneκC]silver(I)} chloride 1,2-dichloroethane hemisolvate Fractional atomic coordinates and isotropic or equivalent isotropic displacement parameters (Å^2^)

**Table:**

	*x*	*y*	*z*	*U*_iso_*/*U*_eq_	Occ. (<1)
Ag1	0.25650 (2)	0.49164 (2)	0.33797 (2)	0.01912 (6)	
Ag2	0.26003 (2)	0.73310 (2)	0.37289 (2)	0.01975 (6)	
Cl1	0.26361 (4)	0.60834 (2)	0.38575 (2)	0.02881 (12)	
Cl2	0.76215 (4)	0.61174 (2)	0.38397 (2)	0.02090 (10)	
N1	0.50114 (15)	0.50958 (8)	0.34948 (7)	0.0197 (4)	
N2	0.45511 (14)	0.42868 (8)	0.38637 (7)	0.0185 (3)	
N3	0.05469 (15)	0.48589 (8)	0.26312 (8)	0.0211 (4)	
N4	0.01433 (15)	0.51172 (8)	0.34141 (7)	0.0193 (4)	
N5	0.45631 (15)	0.76470 (8)	0.30943 (7)	0.0205 (4)	
N6	0.50338 (15)	0.71660 (9)	0.37995 (7)	0.0211 (4)	
N7	0.06974 (15)	0.79873 (9)	0.41778 (8)	0.0225 (4)	
N8	0.01457 (15)	0.72187 (9)	0.37458 (8)	0.0218 (4)	
C1	0.41603 (17)	0.47505 (10)	0.35709 (8)	0.0187 (4)	
C2	0.59158 (18)	0.48536 (10)	0.37375 (9)	0.0224 (4)	
H2	0.660426	0.501508	0.373856	0.027*	
C3	0.56224 (17)	0.43432 (11)	0.39716 (9)	0.0224 (4)	
H3	0.606450	0.407418	0.417178	0.027*	
C4	0.39110 (17)	0.38217 (10)	0.40749 (8)	0.0187 (4)	
C5	0.37793 (19)	0.32912 (11)	0.37902 (9)	0.0249 (5)	
C6	0.3192 (2)	0.28405 (11)	0.40114 (10)	0.0276 (5)	
H6	0.310208	0.247313	0.382528	0.033*	
C7	0.27347 (18)	0.29150 (11)	0.44979 (9)	0.0249 (5)	
C8	0.28657 (17)	0.34547 (11)	0.47676 (8)	0.0229 (4)	
H8	0.254234	0.351120	0.509711	0.027*	
C9	0.34635 (17)	0.39148 (10)	0.45631 (8)	0.0197 (4)	
C10	0.4275 (3)	0.32017 (13)	0.32639 (11)	0.0388 (6)	
H10A	0.393562	0.346391	0.299338	0.058*	
H10B	0.418929	0.278518	0.315097	0.058*	
H10C	0.502468	0.329787	0.330465	0.058*	
C11	0.2123 (2)	0.24108 (13)	0.47354 (11)	0.0361 (6)	
H11A	0.212994	0.245736	0.512502	0.054*	
H11B	0.244511	0.202866	0.464800	0.054*	
H11C	0.139633	0.241978	0.458708	0.054*	
C12	0.3636 (2)	0.44866 (12)	0.48676 (10)	0.0316 (5)	
H12A	0.438467	0.453327	0.496626	0.047*	
H12B	0.323910	0.447659	0.519230	0.047*	
H12C	0.339534	0.482312	0.464308	0.047*	
C13	0.49867 (18)	0.56636 (9)	0.32015 (9)	0.0216 (4)	
H13A	0.550170	0.594040	0.337756	0.026*	
H13B	0.428173	0.584412	0.322128	0.026*	
C14	0.52324 (19)	0.55953 (9)	0.26197 (9)	0.0226 (4)	
C15	0.6231 (2)	0.57206 (12)	0.24564 (11)	0.0322 (5)	
H15	0.676933	0.583082	0.271447	0.039*	
C16	0.6453 (3)	0.56864 (14)	0.19173 (12)	0.0432 (7)	
H16	0.714000	0.577032	0.180893	0.052*	
C17	0.5665 (3)	0.55291 (13)	0.15408 (11)	0.0439 (7)	
H17	0.580819	0.551430	0.117231	0.053*	
C18	0.4673 (3)	0.53941 (13)	0.16999 (11)	0.0397 (6)	
H18	0.413780	0.528103	0.144145	0.048*	
C19	0.4457 (2)	0.54233 (12)	0.22396 (10)	0.0308 (5)	
H19	0.377575	0.532520	0.234804	0.037*	
C20	0.09670 (18)	0.49400 (9)	0.31352 (9)	0.0196 (4)	
C21	−0.07723 (18)	0.51556 (10)	0.30951 (9)	0.0235 (4)	
H21	−0.144391	0.527346	0.320335	0.028*	
C22	−0.0520 (2)	0.49915 (11)	0.25977 (10)	0.0258 (5)	
H22	−0.098055	0.497096	0.228714	0.031*	
C23	0.11352 (18)	0.46358 (10)	0.21996 (9)	0.0226 (4)	
C24	0.1439 (2)	0.50266 (11)	0.18046 (10)	0.0288 (5)	
C25	0.2013 (2)	0.47913 (13)	0.13931 (11)	0.0349 (6)	
H25	0.221595	0.504744	0.111437	0.042*	
C26	0.2293 (2)	0.41914 (14)	0.13811 (11)	0.0353 (6)	
C27	0.1974 (2)	0.38204 (12)	0.17792 (11)	0.0335 (5)	
H27	0.216216	0.341048	0.177229	0.040*	
C28	0.1380 (2)	0.40301 (11)	0.21938 (10)	0.0278 (5)	
C29	0.1175 (3)	0.56839 (13)	0.18214 (13)	0.0449 (7)	
H29A	0.041118	0.573424	0.180233	0.067*	
H29B	0.147185	0.588618	0.151690	0.067*	
H29C	0.147133	0.585707	0.215589	0.067*	
C30	0.2943 (3)	0.39469 (16)	0.09405 (13)	0.0491 (8)	
H30A	0.263823	0.407707	0.059197	0.074*	
H30B	0.294579	0.351007	0.095605	0.074*	
H30C	0.366370	0.409572	0.098869	0.074*	
C31	0.1033 (3)	0.36087 (12)	0.26152 (13)	0.0417 (7)	
H31A	0.144739	0.367921	0.295015	0.062*	
H31B	0.113539	0.319673	0.249783	0.062*	
H31C	0.028789	0.367497	0.267293	0.062*	
C32	0.01990 (18)	0.52358 (10)	0.39944 (8)	0.0210 (4)	
H32A	−0.021671	0.559746	0.406547	0.025*	
H32B	0.093721	0.531671	0.411422	0.025*	
C33	−0.02075 (18)	0.47215 (10)	0.43157 (8)	0.0205 (4)	
C34	0.03972 (19)	0.42105 (11)	0.44112 (10)	0.0286 (5)	
H34	0.105805	0.417284	0.425668	0.034*	
C35	0.0037 (2)	0.37566 (11)	0.47314 (11)	0.0333 (5)	
H35	0.045391	0.341042	0.479654	0.040*	
C36	−0.0929 (2)	0.38064 (11)	0.49565 (9)	0.0300 (5)	
H36	−0.116953	0.349696	0.517853	0.036*	
C37	−0.1542 (2)	0.43094 (11)	0.48568 (9)	0.0261 (5)	
H37	−0.220860	0.434232	0.500615	0.031*	
C38	−0.11791 (19)	0.47658 (10)	0.45372 (8)	0.0222 (4)	
H38	−0.159971	0.511025	0.447036	0.027*	
C39	0.41754 (18)	0.73962 (9)	0.35341 (9)	0.0197 (4)	
C40	0.56418 (19)	0.75694 (12)	0.30829 (10)	0.0260 (5)	
H40	0.608404	0.770335	0.281377	0.031*	
C41	0.59329 (19)	0.72658 (11)	0.35306 (10)	0.0251 (5)	
H41	0.662423	0.714382	0.364024	0.030*	
C42	0.38975 (17)	0.79130 (10)	0.26771 (8)	0.0208 (4)	
C43	0.3758 (2)	0.85258 (11)	0.26738 (9)	0.0254 (5)	
C44	0.3079 (2)	0.87696 (11)	0.22738 (10)	0.0299 (5)	
H44	0.296964	0.918877	0.226316	0.036*	
C45	0.2562 (2)	0.84111 (12)	0.18926 (9)	0.0302 (5)	
C46	0.2743 (2)	0.77998 (12)	0.19039 (9)	0.0278 (5)	
H46	0.240336	0.755401	0.163841	0.033*	
C47	0.34106 (18)	0.75395 (11)	0.22945 (9)	0.0232 (4)	
C48	0.4312 (3)	0.89177 (12)	0.30863 (11)	0.0382 (6)	
H48A	0.507127	0.889639	0.304648	0.057*	
H48B	0.415216	0.878140	0.344499	0.057*	
H48C	0.407498	0.933070	0.303623	0.057*	
C49	0.1799 (3)	0.86776 (16)	0.14784 (12)	0.0445 (7)	
H49A	0.178161	0.843244	0.115376	0.067*	
H49B	0.202271	0.908328	0.139285	0.067*	
H49C	0.109750	0.869045	0.161915	0.067*	
C50	0.3594 (2)	0.68754 (11)	0.23057 (11)	0.0329 (5)	
H50A	0.331122	0.670652	0.262933	0.049*	
H50B	0.434881	0.679496	0.230600	0.049*	
H50C	0.324084	0.669276	0.198900	0.049*	
C51	0.49911 (19)	0.68416 (11)	0.43059 (9)	0.0263 (5)	
H51A	0.566747	0.663624	0.438797	0.032*	0.446 (13)
H51B	0.443045	0.653621	0.427603	0.032*	0.446 (13)
H51C	0.568487	0.665685	0.439051	0.032*	0.554 (13)
H51D	0.447191	0.651509	0.425834	0.032*	0.554 (13)
C52A	0.477 (2)	0.7281 (8)	0.4758 (8)	0.0306 (19)	0.446 (13)
C53A	0.4191 (10)	0.7132 (6)	0.5196 (5)	0.043 (2)	0.446 (13)
H53A	0.385766	0.675254	0.519121	0.052*	0.446 (13)
C54A	0.4068 (10)	0.7489 (7)	0.5632 (4)	0.048 (2)	0.446 (13)
H54A	0.364780	0.736519	0.591374	0.058*	0.446 (13)
C55A	0.4569 (10)	0.8035 (5)	0.5651 (4)	0.049 (2)	0.446 (13)
H55A	0.451417	0.828630	0.595344	0.059*	0.446 (13)
C56A	0.5153 (10)	0.8216 (5)	0.5228 (4)	0.046 (2)	0.446 (13)
H56A	0.550281	0.858996	0.524217	0.055*	0.446 (13)
C57A	0.5227 (16)	0.7848 (6)	0.4783 (5)	0.034 (2)	0.446 (13)
H57A	0.559839	0.798675	0.448817	0.041*	0.446 (13)
C52B	0.4708 (18)	0.7213 (6)	0.4769 (6)	0.0305 (16)	0.554 (13)
C53B	0.3990 (8)	0.6978 (4)	0.5104 (3)	0.0369 (16)	0.554 (13)
H53B	0.366095	0.660689	0.502215	0.044*	0.554 (13)
C54B	0.3752 (8)	0.7288 (4)	0.5560 (3)	0.0461 (17)	0.554 (13)
H54B	0.327336	0.712149	0.579657	0.055*	0.554 (13)
C55B	0.4197 (8)	0.7834 (5)	0.5675 (3)	0.0460 (18)	0.554 (13)
H55B	0.404442	0.803825	0.599372	0.055*	0.554 (13)
C56B	0.4870 (8)	0.8083 (4)	0.5322 (4)	0.0446 (18)	0.554 (13)
H56B	0.514633	0.847130	0.538734	0.054*	0.554 (13)
C57B	0.5144 (13)	0.7767 (6)	0.4874 (5)	0.0384 (19)	0.554 (13)
H57B	0.562989	0.793219	0.463944	0.046*	0.554 (13)
C58	0.10313 (17)	0.75233 (10)	0.38836 (9)	0.0210 (4)	
C59	−0.03804 (19)	0.79686 (12)	0.42260 (11)	0.0294 (5)	
H59	−0.079278	0.824304	0.441382	0.035*	
C60	−0.07233 (19)	0.74830 (12)	0.39529 (11)	0.0291 (5)	
H60	−0.142830	0.734764	0.391062	0.035*	
C61	0.13917 (18)	0.84400 (11)	0.43993 (10)	0.0236 (4)	
C62	0.1780 (2)	0.88649 (11)	0.40505 (10)	0.0275 (5)	
C63	0.2491 (2)	0.92812 (12)	0.42635 (11)	0.0336 (5)	
H63	0.276448	0.957406	0.403441	0.040*	
C64	0.2815 (2)	0.92812 (13)	0.48039 (12)	0.0360 (6)	
C65	0.2385 (2)	0.88693 (13)	0.51405 (10)	0.0354 (6)	
H65	0.257943	0.887981	0.551219	0.042*	
C66	0.1669 (2)	0.84359 (12)	0.49453 (10)	0.0283 (5)	
C67	0.1434 (2)	0.88746 (12)	0.34665 (11)	0.0364 (6)	
H67A	0.165768	0.850568	0.329602	0.055*	
H67B	0.175026	0.921770	0.329369	0.055*	
H67C	0.066737	0.890663	0.342947	0.055*	
C68	0.3635 (3)	0.97202 (18)	0.50161 (16)	0.0563 (9)	
H68A	0.369911	0.969524	0.540751	0.084*	
H68B	0.342517	1.012538	0.490904	0.084*	
H68C	0.431151	0.962457	0.486940	0.084*	
C69	0.1239 (3)	0.79815 (15)	0.53190 (11)	0.0406 (6)	
H69A	0.047369	0.802099	0.531808	0.061*	
H69B	0.154248	0.804813	0.568210	0.061*	
H69C	0.141986	0.757970	0.520017	0.061*	
C70	0.00879 (18)	0.66948 (10)	0.33935 (9)	0.0236 (4)	
H70A	−0.041346	0.640322	0.353202	0.028*	
H70B	0.078601	0.650183	0.339519	0.028*	
C71	−0.02584 (19)	0.68623 (10)	0.28266 (9)	0.0235 (4)	
C72	−0.1306 (2)	0.67892 (12)	0.26496 (11)	0.0337 (5)	
H72	−0.179734	0.663474	0.288622	0.040*	
C73	−0.1635 (2)	0.69413 (14)	0.21289 (12)	0.0392 (6)	
H73	−0.235068	0.689455	0.201207	0.047*	
C74	−0.0922 (3)	0.71609 (13)	0.17798 (11)	0.0375 (6)	
H74	−0.114459	0.725929	0.142237	0.045*	
C75	0.0119 (2)	0.72364 (14)	0.19547 (11)	0.0377 (6)	
H75	0.060788	0.739088	0.171711	0.045*	
C76	0.0448 (2)	0.70883 (12)	0.24728 (11)	0.0312 (5)	
H76	0.116333	0.714105	0.258851	0.037*	
Cl3	0.0888 (3)	0.4284 (2)	−0.0300 (3)	0.0605 (14)	0.423 (16)
C77	0.0185 (11)	0.4734 (6)	0.0150 (5)	0.079 (3)	0.423 (16)
H77A	−0.041789	0.450984	0.028195	0.095*	0.423 (16)
H77B	0.065000	0.485270	0.046163	0.095*	0.423 (16)
Cl3B	0.0857 (6)	0.4297 (3)	−0.0383 (4)	0.123 (3)	0.577 (16)
C77B	0.0149 (8)	0.4967 (4)	−0.0277 (4)	0.075 (3)	0.577 (16)
H77C	0.058576	0.531196	−0.037272	0.090*	0.577 (16)
H77D	−0.049328	0.497044	−0.051768	0.090*	0.577 (16)

### µ-Chlorido-bis{[1-benzyl-3-(2,4,6-trimethylphenyl)imidazol-2-ylideneκC]silver(I)} chloride 1,2-dichloroethane hemisolvate Atomic displacement parameters (Å^2^)

**Table:**

	*U*^11^	*U*^22^	*U*^33^	*U*^12^	*U*^13^	*U*^23^
Ag1	0.01787 (9)	0.02148 (9)	0.01785 (8)	−0.00119 (5)	−0.00058 (6)	0.00167 (5)
Ag2	0.01887 (9)	0.02164 (9)	0.01876 (8)	−0.00295 (5)	0.00135 (6)	0.00269 (5)
Cl1	0.0251 (3)	0.0186 (2)	0.0431 (3)	−0.00207 (19)	0.0057 (2)	−0.0027 (2)
Cl2	0.0202 (2)	0.0215 (2)	0.0210 (2)	−0.00032 (17)	0.00176 (17)	0.00138 (18)
N1	0.0206 (9)	0.0200 (9)	0.0186 (8)	−0.0013 (7)	0.0021 (7)	−0.0013 (7)
N2	0.0192 (8)	0.0203 (8)	0.0163 (8)	−0.0014 (7)	0.0026 (6)	−0.0014 (6)
N3	0.0221 (9)	0.0216 (9)	0.0193 (9)	−0.0009 (7)	−0.0013 (7)	−0.0043 (7)
N4	0.0207 (9)	0.0189 (9)	0.0180 (8)	−0.0017 (7)	−0.0018 (7)	−0.0020 (6)
N5	0.0218 (9)	0.0230 (9)	0.0167 (8)	−0.0009 (7)	−0.0003 (7)	0.0050 (7)
N6	0.0218 (9)	0.0224 (9)	0.0186 (8)	−0.0018 (7)	−0.0015 (7)	0.0042 (7)
N7	0.0208 (9)	0.0227 (9)	0.0239 (9)	−0.0029 (7)	0.0016 (7)	0.0014 (7)
N8	0.0207 (9)	0.0217 (9)	0.0231 (9)	−0.0038 (7)	0.0006 (7)	0.0023 (7)
C1	0.0194 (10)	0.0196 (9)	0.0173 (9)	−0.0007 (8)	0.0018 (7)	−0.0020 (8)
C2	0.0193 (10)	0.0262 (11)	0.0216 (10)	−0.0019 (8)	0.0015 (8)	−0.0010 (8)
C3	0.0196 (10)	0.0264 (11)	0.0212 (10)	0.0015 (8)	0.0011 (8)	0.0019 (8)
C4	0.0185 (9)	0.0223 (10)	0.0151 (9)	−0.0022 (8)	−0.0002 (7)	0.0024 (8)
C5	0.0288 (11)	0.0280 (12)	0.0182 (10)	−0.0054 (9)	0.0045 (8)	−0.0031 (8)
C6	0.0326 (12)	0.0275 (11)	0.0229 (11)	−0.0087 (10)	0.0027 (9)	−0.0045 (9)
C7	0.0242 (11)	0.0288 (12)	0.0218 (10)	−0.0055 (9)	0.0013 (8)	0.0062 (9)
C8	0.0216 (10)	0.0331 (12)	0.0140 (9)	0.0025 (9)	0.0021 (8)	0.0041 (8)
C9	0.0197 (10)	0.0239 (10)	0.0155 (9)	0.0024 (8)	−0.0006 (7)	0.0014 (8)
C10	0.0556 (17)	0.0373 (14)	0.0249 (12)	−0.0169 (13)	0.0163 (12)	−0.0115 (10)
C11	0.0389 (14)	0.0371 (14)	0.0328 (13)	−0.0119 (11)	0.0071 (11)	0.0077 (11)
C12	0.0427 (14)	0.0291 (12)	0.0238 (11)	−0.0005 (11)	0.0105 (10)	−0.0060 (9)
C13	0.0251 (10)	0.0159 (9)	0.0241 (10)	−0.0002 (8)	0.0040 (8)	0.0002 (8)
C14	0.0310 (11)	0.0143 (9)	0.0227 (10)	0.0016 (8)	0.0026 (9)	0.0019 (8)
C15	0.0365 (13)	0.0322 (13)	0.0283 (12)	−0.0079 (10)	0.0061 (10)	−0.0003 (10)
C16	0.0563 (18)	0.0401 (15)	0.0350 (14)	−0.0101 (13)	0.0198 (13)	0.0008 (11)
C17	0.079 (2)	0.0317 (14)	0.0220 (12)	0.0025 (14)	0.0092 (13)	0.0018 (10)
C18	0.0587 (18)	0.0338 (14)	0.0253 (12)	0.0068 (12)	−0.0084 (12)	−0.0040 (10)
C19	0.0348 (13)	0.0272 (12)	0.0299 (12)	0.0045 (10)	−0.0032 (10)	−0.0007 (9)
C20	0.0221 (10)	0.0179 (9)	0.0186 (10)	−0.0012 (8)	−0.0008 (8)	−0.0007 (7)
C21	0.0219 (11)	0.0250 (11)	0.0234 (11)	0.0014 (8)	−0.0019 (8)	−0.0046 (8)
C22	0.0249 (11)	0.0279 (11)	0.0239 (11)	0.0025 (9)	−0.0061 (9)	−0.0059 (9)
C23	0.0255 (11)	0.0236 (11)	0.0185 (10)	−0.0013 (8)	−0.0008 (8)	−0.0066 (8)
C24	0.0364 (13)	0.0247 (12)	0.0255 (11)	−0.0018 (10)	0.0028 (10)	−0.0032 (9)
C25	0.0439 (15)	0.0361 (13)	0.0255 (12)	−0.0027 (12)	0.0087 (11)	−0.0026 (10)
C26	0.0382 (14)	0.0398 (14)	0.0281 (12)	0.0025 (11)	0.0053 (10)	−0.0125 (11)
C27	0.0399 (14)	0.0252 (12)	0.0355 (13)	0.0042 (10)	0.0030 (11)	−0.0112 (10)
C28	0.0325 (12)	0.0234 (11)	0.0274 (11)	0.0000 (9)	0.0010 (9)	−0.0035 (9)
C29	0.066 (2)	0.0253 (13)	0.0454 (16)	0.0043 (13)	0.0186 (14)	0.0058 (11)
C30	0.059 (2)	0.0523 (18)	0.0375 (15)	0.0052 (15)	0.0176 (14)	−0.0151 (13)
C31	0.0599 (19)	0.0229 (12)	0.0432 (15)	0.0011 (12)	0.0132 (13)	0.0017 (11)
C32	0.0252 (10)	0.0219 (10)	0.0159 (9)	−0.0031 (8)	0.0002 (8)	−0.0030 (8)
C33	0.0249 (10)	0.0194 (10)	0.0167 (9)	−0.0018 (8)	−0.0035 (8)	−0.0031 (8)
C34	0.0254 (11)	0.0271 (12)	0.0332 (12)	0.0028 (9)	0.0001 (9)	0.0001 (9)
C35	0.0389 (14)	0.0229 (12)	0.0373 (13)	0.0044 (10)	−0.0066 (11)	0.0042 (10)
C36	0.0450 (14)	0.0243 (11)	0.0201 (10)	−0.0066 (10)	−0.0043 (9)	0.0030 (8)
C37	0.0315 (12)	0.0289 (12)	0.0180 (10)	−0.0058 (9)	0.0012 (8)	−0.0027 (8)
C38	0.0277 (11)	0.0220 (10)	0.0164 (9)	0.0007 (9)	−0.0026 (8)	−0.0024 (8)
C39	0.0233 (10)	0.0179 (9)	0.0177 (9)	−0.0026 (8)	0.0000 (8)	0.0027 (7)
C40	0.0223 (11)	0.0337 (12)	0.0225 (10)	0.0006 (9)	0.0043 (8)	0.0063 (9)
C41	0.0204 (10)	0.0299 (12)	0.0250 (11)	0.0014 (9)	0.0012 (9)	0.0042 (9)
C42	0.0218 (10)	0.0237 (11)	0.0170 (9)	0.0027 (8)	0.0027 (8)	0.0063 (8)
C43	0.0325 (12)	0.0228 (11)	0.0214 (10)	0.0008 (9)	0.0069 (9)	0.0044 (8)
C44	0.0409 (14)	0.0239 (11)	0.0257 (11)	0.0110 (10)	0.0103 (10)	0.0107 (9)
C45	0.0311 (12)	0.0389 (13)	0.0211 (10)	0.0111 (10)	0.0066 (9)	0.0138 (9)
C46	0.0289 (12)	0.0366 (13)	0.0178 (10)	0.0018 (10)	0.0010 (9)	0.0039 (9)
C47	0.0237 (11)	0.0263 (11)	0.0197 (10)	0.0005 (9)	0.0027 (8)	0.0028 (8)
C48	0.0562 (17)	0.0247 (12)	0.0334 (13)	−0.0009 (12)	−0.0009 (12)	−0.0019 (10)
C49	0.0427 (16)	0.0572 (19)	0.0333 (13)	0.0170 (14)	0.0003 (12)	0.0190 (13)
C50	0.0403 (14)	0.0250 (12)	0.0326 (12)	0.0009 (10)	−0.0048 (10)	−0.0004 (9)
C51	0.0278 (11)	0.0291 (11)	0.0215 (10)	−0.0017 (9)	−0.0034 (8)	0.0104 (9)
C52A	0.035 (4)	0.040 (4)	0.017 (3)	0.011 (3)	0.003 (3)	0.013 (3)
C53A	0.054 (4)	0.045 (4)	0.030 (3)	0.009 (3)	0.005 (3)	0.011 (3)
C54A	0.064 (4)	0.051 (5)	0.031 (3)	0.003 (4)	0.013 (3)	0.009 (4)
C55A	0.067 (4)	0.048 (4)	0.033 (3)	0.011 (3)	0.008 (3)	0.004 (3)
C56A	0.060 (4)	0.047 (4)	0.030 (4)	0.007 (3)	0.005 (3)	−0.001 (3)
C57A	0.045 (4)	0.037 (4)	0.020 (4)	0.005 (3)	−0.002 (3)	0.002 (3)
C52B	0.034 (3)	0.035 (3)	0.023 (3)	0.009 (3)	−0.005 (2)	0.012 (3)
C53B	0.050 (3)	0.039 (4)	0.023 (3)	0.008 (3)	0.009 (2)	0.012 (2)
C54B	0.066 (4)	0.042 (4)	0.032 (3)	0.011 (3)	0.016 (3)	0.009 (3)
C55B	0.065 (4)	0.048 (4)	0.026 (2)	0.011 (3)	0.013 (3)	0.003 (3)
C56B	0.058 (4)	0.049 (4)	0.028 (3)	0.010 (3)	0.009 (3)	−0.001 (3)
C57B	0.045 (3)	0.045 (4)	0.025 (4)	0.006 (3)	0.002 (3)	0.003 (3)
C58	0.0210 (10)	0.0219 (10)	0.0199 (10)	−0.0034 (8)	−0.0002 (8)	0.0052 (8)
C59	0.0227 (11)	0.0292 (12)	0.0367 (13)	−0.0021 (9)	0.0056 (9)	−0.0040 (10)
C60	0.0191 (11)	0.0318 (12)	0.0364 (13)	−0.0036 (9)	0.0018 (9)	−0.0033 (10)
C61	0.0207 (10)	0.0233 (11)	0.0269 (11)	−0.0033 (8)	0.0008 (8)	−0.0006 (9)
C62	0.0298 (12)	0.0219 (11)	0.0311 (12)	−0.0008 (9)	0.0045 (9)	0.0023 (9)
C63	0.0377 (13)	0.0260 (12)	0.0377 (13)	−0.0094 (10)	0.0077 (11)	0.0004 (10)
C64	0.0344 (13)	0.0350 (13)	0.0386 (14)	−0.0116 (11)	0.0009 (11)	−0.0089 (11)
C65	0.0385 (14)	0.0420 (15)	0.0255 (12)	−0.0070 (11)	−0.0012 (10)	−0.0051 (11)
C66	0.0304 (12)	0.0298 (12)	0.0248 (11)	−0.0041 (10)	0.0024 (9)	−0.0003 (9)
C67	0.0476 (16)	0.0310 (13)	0.0304 (13)	−0.0071 (11)	−0.0003 (11)	0.0086 (10)
C68	0.059 (2)	0.055 (2)	0.0547 (19)	−0.0323 (17)	0.0014 (16)	−0.0141 (16)
C69	0.0488 (16)	0.0467 (16)	0.0263 (12)	−0.0120 (13)	0.0010 (11)	0.0054 (11)
C70	0.0249 (11)	0.0182 (10)	0.0274 (11)	−0.0038 (8)	−0.0019 (8)	0.0008 (8)
C71	0.0277 (11)	0.0162 (10)	0.0265 (11)	−0.0009 (8)	−0.0009 (9)	−0.0006 (8)
C72	0.0336 (13)	0.0342 (13)	0.0326 (13)	−0.0116 (10)	−0.0044 (10)	0.0083 (10)
C73	0.0380 (14)	0.0444 (15)	0.0339 (13)	−0.0128 (12)	−0.0113 (11)	0.0071 (12)
C74	0.0492 (16)	0.0386 (14)	0.0241 (11)	−0.0001 (12)	−0.0034 (11)	0.0009 (10)
C75	0.0403 (15)	0.0445 (15)	0.0291 (13)	0.0027 (12)	0.0108 (11)	0.0019 (11)
C76	0.0282 (12)	0.0347 (13)	0.0310 (12)	0.0021 (10)	0.0042 (10)	−0.0018 (10)
Cl3	0.0393 (18)	0.0433 (19)	0.099 (3)	−0.0002 (12)	0.0088 (14)	0.0211 (18)
C77	0.081 (6)	0.067 (6)	0.089 (6)	0.011 (5)	0.006 (5)	0.011 (5)
Cl3B	0.136 (5)	0.096 (4)	0.137 (4)	−0.012 (3)	0.015 (3)	−0.016 (3)
C77B	0.070 (5)	0.066 (5)	0.089 (5)	0.010 (4)	0.001 (4)	0.028 (4)

### µ-Chlorido-bis{[1-benzyl-3-(2,4,6-trimethylphenyl)imidazol-2-ylideneκC]silver(I)} chloride 1,2-dichloroethane hemisolvate Geometric parameters (Å, º)

**Table:**

Ag1—Cl1	2.8755 (6)	C36—C37	1.388 (4)
Ag1—C1	2.099 (2)	C37—H37	0.9500
Ag1—C20	2.098 (2)	C37—C38	1.393 (3)
Ag2—Cl1	2.8149 (6)	C38—H38	0.9500
Ag2—C39	2.098 (2)	C40—H40	0.9500
Ag2—C58	2.104 (2)	C40—C41	1.346 (3)
N1—C1	1.355 (3)	C41—H41	0.9500
N1—C2	1.386 (3)	C42—C43	1.385 (3)
N1—C13	1.469 (3)	C42—C47	1.393 (3)
N2—C1	1.351 (3)	C43—C44	1.400 (4)
N2—C3	1.385 (3)	C43—C48	1.503 (4)
N2—C4	1.442 (3)	C44—H44	0.9500
N3—C20	1.356 (3)	C44—C45	1.387 (4)
N3—C22	1.392 (3)	C45—C46	1.390 (4)
N3—C23	1.437 (3)	C45—C49	1.507 (3)
N4—C20	1.352 (3)	C46—H46	0.9500
N4—C21	1.382 (3)	C46—C47	1.390 (3)
N4—C32	1.474 (3)	C47—C50	1.507 (3)
N5—C39	1.353 (3)	C48—H48A	0.9800
N5—C40	1.389 (3)	C48—H48B	0.9800
N5—C42	1.440 (3)	C48—H48C	0.9800
N6—C39	1.353 (3)	C49—H49A	0.9800
N6—C41	1.379 (3)	C49—H49B	0.9800
N6—C51	1.465 (3)	C49—H49C	0.9800
N7—C58	1.356 (3)	C50—H50A	0.9800
N7—C59	1.388 (3)	C50—H50B	0.9800
N7—C61	1.439 (3)	C50—H50C	0.9800
N8—C58	1.349 (3)	C51—H51A	0.9900
N8—C60	1.382 (3)	C51—H51B	0.9900
N8—C70	1.468 (3)	C51—H51C	0.9900
C2—H2	0.9500	C51—H51D	0.9900
C2—C3	1.348 (3)	C51—C52A	1.538 (10)
C3—H3	0.9500	C51—C52B	1.487 (8)
C4—C5	1.392 (3)	C52A—C53A	1.394 (11)
C4—C9	1.391 (3)	C52A—C57A	1.399 (11)
C5—C6	1.390 (3)	C53A—H53A	0.9500
C5—C10	1.505 (3)	C53A—C54A	1.371 (12)
C6—H6	0.9500	C54A—H54A	0.9500
C6—C7	1.388 (3)	C54A—C55A	1.379 (12)
C7—C8	1.391 (4)	C55A—H55A	0.9500
C7—C11	1.513 (3)	C55A—C56A	1.387 (11)
C8—H8	0.9500	C56A—H56A	0.9500
C8—C9	1.396 (3)	C56A—C57A	1.392 (11)
C9—C12	1.501 (3)	C57A—H57A	0.9500
C10—H10A	0.9800	C52B—C53B	1.378 (10)
C10—H10B	0.9800	C52B—C57B	1.382 (10)
C10—H10C	0.9800	C53B—H53B	0.9500
C11—H11A	0.9800	C53B—C54B	1.383 (9)
C11—H11B	0.9800	C54B—H54B	0.9500
C11—H11C	0.9800	C54B—C55B	1.372 (10)
C12—H12A	0.9800	C55B—H55B	0.9500
C12—H12B	0.9800	C55B—C56B	1.381 (10)
C12—H12C	0.9800	C56B—H56B	0.9500
C13—H13A	0.9900	C56B—C57B	1.387 (9)
C13—H13B	0.9900	C57B—H57B	0.9500
C13—C14	1.513 (3)	C59—H59	0.9500
C14—C15	1.387 (4)	C59—C60	1.346 (4)
C14—C19	1.390 (4)	C60—H60	0.9500
C15—H15	0.9500	C61—C62	1.400 (3)
C15—C16	1.395 (4)	C61—C66	1.393 (3)
C16—H16	0.9500	C62—C63	1.388 (4)
C16—C17	1.386 (5)	C62—C67	1.504 (4)
C17—H17	0.9500	C63—H63	0.9500
C17—C18	1.381 (5)	C63—C64	1.393 (4)
C18—H18	0.9500	C64—C65	1.382 (4)
C18—C19	1.394 (4)	C64—C68	1.512 (4)
C19—H19	0.9500	C65—H65	0.9500
C21—H21	0.9500	C65—C66	1.404 (4)
C21—C22	1.353 (3)	C66—C69	1.506 (4)
C22—H22	0.9500	C67—H67A	0.9800
C23—C24	1.391 (4)	C67—H67B	0.9800
C23—C28	1.393 (3)	C67—H67C	0.9800
C24—C25	1.398 (4)	C68—H68A	0.9800
C24—C29	1.512 (4)	C68—H68B	0.9800
C25—H25	0.9500	C68—H68C	0.9800
C25—C26	1.392 (4)	C69—H69A	0.9800
C26—C27	1.376 (4)	C69—H69B	0.9800
C26—C30	1.517 (4)	C69—H69C	0.9800
C27—H27	0.9500	C70—H70A	0.9900
C27—C28	1.398 (4)	C70—H70B	0.9900
C28—C31	1.500 (4)	C70—C71	1.511 (3)
C29—H29A	0.9800	C71—C72	1.396 (4)
C29—H29B	0.9800	C71—C76	1.392 (4)
C29—H29C	0.9800	C72—H72	0.9500
C30—H30A	0.9800	C72—C73	1.390 (4)
C30—H30B	0.9800	C73—H73	0.9500
C30—H30C	0.9800	C73—C74	1.385 (4)
C31—H31A	0.9800	C74—H74	0.9500
C31—H31B	0.9800	C74—C75	1.386 (5)
C31—H31C	0.9800	C75—H75	0.9500
C32—H32A	0.9900	C75—C76	1.382 (4)
C32—H32B	0.9900	C76—H76	0.9500
C32—C33	1.513 (3)	Cl3—C77	1.790 (14)
C33—C34	1.395 (3)	C77—C77^i^	1.47 (3)
C33—C38	1.388 (3)	C77—H77A	0.9900
C34—H34	0.9500	C77—H77B	0.9900
C34—C35	1.388 (4)	Cl3B—C77B	1.780 (11)
C35—H35	0.9500	C77B—C77B^i^	1.47 (2)
C35—C36	1.386 (4)	C77B—H77C	0.9900
C36—H36	0.9500	C77B—H77D	0.9900
			
C1—Ag1—Cl1	93.30 (6)	N5—C40—H40	127.0
C20—Ag1—Cl1	96.14 (6)	C41—C40—N5	106.0 (2)
C20—Ag1—C1	170.55 (8)	C41—C40—H40	127.0
C39—Ag2—Cl1	94.92 (6)	N6—C41—H41	126.6
C39—Ag2—C58	163.97 (8)	C40—C41—N6	106.8 (2)
C58—Ag2—Cl1	101.09 (6)	C40—C41—H41	126.6
Ag2—Cl1—Ag1	148.90 (2)	C43—C42—N5	119.1 (2)
C1—N1—C2	111.69 (19)	C43—C42—C47	122.6 (2)
C1—N1—C13	124.68 (19)	C47—C42—N5	118.3 (2)
C2—N1—C13	123.62 (19)	C42—C43—C44	117.8 (2)
C1—N2—C3	111.56 (18)	C42—C43—C48	121.3 (2)
C1—N2—C4	123.70 (18)	C44—C43—C48	120.9 (2)
C3—N2—C4	124.53 (19)	C43—C44—H44	119.4
C20—N3—C22	111.36 (19)	C45—C44—C43	121.3 (2)
C20—N3—C23	123.3 (2)	C45—C44—H44	119.4
C22—N3—C23	125.28 (19)	C44—C45—C46	119.0 (2)
C20—N4—C21	112.12 (19)	C44—C45—C49	120.6 (3)
C20—N4—C32	124.30 (19)	C46—C45—C49	120.4 (3)
C21—N4—C32	123.54 (19)	C45—C46—H46	119.2
C39—N5—C40	111.75 (19)	C45—C46—C47	121.6 (2)
C39—N5—C42	122.33 (19)	C47—C46—H46	119.2
C40—N5—C42	125.72 (19)	C42—C47—C50	121.2 (2)
C39—N6—C41	111.82 (19)	C46—C47—C42	117.7 (2)
C39—N6—C51	123.2 (2)	C46—C47—C50	121.1 (2)
C41—N6—C51	125.0 (2)	C43—C48—H48A	109.5
C58—N7—C59	111.4 (2)	C43—C48—H48B	109.5
C58—N7—C61	123.06 (19)	C43—C48—H48C	109.5
C59—N7—C61	125.5 (2)	H48A—C48—H48B	109.5
C58—N8—C60	111.4 (2)	H48A—C48—H48C	109.5
C58—N8—C70	125.0 (2)	H48B—C48—H48C	109.5
C60—N8—C70	123.5 (2)	C45—C49—H49A	109.5
N1—C1—Ag1	129.90 (16)	C45—C49—H49B	109.5
N2—C1—Ag1	125.80 (16)	C45—C49—H49C	109.5
N2—C1—N1	103.89 (18)	H49A—C49—H49B	109.5
N1—C2—H2	126.9	H49A—C49—H49C	109.5
C3—C2—N1	106.2 (2)	H49B—C49—H49C	109.5
C3—C2—H2	126.9	C47—C50—H50A	109.5
N2—C3—H3	126.7	C47—C50—H50B	109.5
C2—C3—N2	106.7 (2)	C47—C50—H50C	109.5
C2—C3—H3	126.7	H50A—C50—H50B	109.5
C5—C4—N2	119.04 (19)	H50A—C50—H50C	109.5
C9—C4—N2	118.7 (2)	H50B—C50—H50C	109.5
C9—C4—C5	122.2 (2)	N6—C51—H51A	109.8
C4—C5—C10	121.1 (2)	N6—C51—H51B	109.8
C6—C5—C4	118.0 (2)	N6—C51—H51C	108.6
C6—C5—C10	120.8 (2)	N6—C51—H51D	108.6
C5—C6—H6	119.2	N6—C51—C52A	109.6 (9)
C7—C6—C5	121.5 (2)	N6—C51—C52B	114.7 (8)
C7—C6—H6	119.2	H51A—C51—H51B	108.2
C6—C7—C8	119.0 (2)	H51C—C51—H51D	107.6
C6—C7—C11	120.3 (2)	C52A—C51—H51A	109.8
C8—C7—C11	120.7 (2)	C52A—C51—H51B	109.8
C7—C8—H8	119.4	C52B—C51—H51C	108.6
C7—C8—C9	121.2 (2)	C52B—C51—H51D	108.6
C9—C8—H8	119.4	C53A—C52A—C51	123.3 (10)
C4—C9—C8	118.0 (2)	C53A—C52A—C57A	114.9 (9)
C4—C9—C12	121.2 (2)	C57A—C52A—C51	121.5 (10)
C8—C9—C12	120.8 (2)	C52A—C53A—H53A	117.5
C5—C10—H10A	109.5	C54A—C53A—C52A	125.0 (10)
C5—C10—H10B	109.5	C54A—C53A—H53A	117.5
C5—C10—H10C	109.5	C53A—C54A—H54A	120.9
H10A—C10—H10B	109.5	C53A—C54A—C55A	118.2 (9)
H10A—C10—H10C	109.5	C55A—C54A—H54A	120.9
H10B—C10—H10C	109.5	C54A—C55A—H55A	120.0
C7—C11—H11A	109.5	C54A—C55A—C56A	120.0 (8)
C7—C11—H11B	109.5	C56A—C55A—H55A	120.0
C7—C11—H11C	109.5	C55A—C56A—H56A	120.0
H11A—C11—H11B	109.5	C55A—C56A—C57A	119.9 (9)
H11A—C11—H11C	109.5	C57A—C56A—H56A	120.0
H11B—C11—H11C	109.5	C52A—C57A—H57A	119.1
C9—C12—H12A	109.5	C56A—C57A—C52A	121.8 (10)
C9—C12—H12B	109.5	C56A—C57A—H57A	119.1
C9—C12—H12C	109.5	C53B—C52B—C51	117.2 (8)
H12A—C12—H12B	109.5	C53B—C52B—C57B	120.1 (7)
H12A—C12—H12C	109.5	C57B—C52B—C51	122.6 (9)
H12B—C12—H12C	109.5	C52B—C53B—H53B	120.3
N1—C13—H13A	108.9	C52B—C53B—C54B	119.5 (7)
N1—C13—H13B	108.9	C54B—C53B—H53B	120.3
N1—C13—C14	113.15 (18)	C53B—C54B—H54B	119.6
H13A—C13—H13B	107.8	C55B—C54B—C53B	120.9 (7)
C14—C13—H13A	108.9	C55B—C54B—H54B	119.6
C14—C13—H13B	108.9	C54B—C55B—H55B	120.3
C15—C14—C13	120.2 (2)	C54B—C55B—C56B	119.4 (6)
C15—C14—C19	119.1 (2)	C56B—C55B—H55B	120.3
C19—C14—C13	120.7 (2)	C55B—C56B—H56B	119.9
C14—C15—H15	119.6	C55B—C56B—C57B	120.2 (8)
C14—C15—C16	120.7 (3)	C57B—C56B—H56B	119.9
C16—C15—H15	119.6	C52B—C57B—C56B	119.7 (9)
C15—C16—H16	120.2	C52B—C57B—H57B	120.1
C17—C16—C15	119.6 (3)	C56B—C57B—H57B	120.1
C17—C16—H16	120.2	N7—C58—Ag2	125.92 (16)
C16—C17—H17	119.9	N8—C58—Ag2	129.91 (18)
C18—C17—C16	120.2 (3)	N8—C58—N7	104.11 (19)
C18—C17—H17	119.9	N7—C59—H59	127.0
C17—C18—H18	120.0	C60—C59—N7	106.1 (2)
C17—C18—C19	120.1 (3)	C60—C59—H59	127.0
C19—C18—H18	120.0	N8—C60—H60	126.5
C14—C19—C18	120.3 (3)	C59—C60—N8	106.9 (2)
C14—C19—H19	119.8	C59—C60—H60	126.5
C18—C19—H19	119.8	C62—C61—N7	118.1 (2)
N3—C20—Ag1	126.71 (17)	C66—C61—N7	119.5 (2)
N4—C20—Ag1	128.83 (16)	C66—C61—C62	122.4 (2)
N4—C20—N3	103.88 (19)	C61—C62—C67	121.3 (2)
N4—C21—H21	126.9	C63—C62—C61	117.6 (2)
C22—C21—N4	106.2 (2)	C63—C62—C67	121.0 (2)
C22—C21—H21	126.9	C62—C63—H63	119.1
N3—C22—H22	126.8	C62—C63—C64	121.8 (2)
C21—C22—N3	106.4 (2)	C64—C63—H63	119.1
C21—C22—H22	126.8	C63—C64—C68	120.3 (3)
C24—C23—N3	119.4 (2)	C65—C64—C63	119.0 (2)
C24—C23—C28	122.3 (2)	C65—C64—C68	120.7 (3)
C28—C23—N3	118.2 (2)	C64—C65—H65	119.3
C23—C24—C25	117.6 (2)	C64—C65—C66	121.5 (2)
C23—C24—C29	121.5 (2)	C66—C65—H65	119.3
C25—C24—C29	120.9 (3)	C61—C66—C65	117.6 (2)
C24—C25—H25	119.1	C61—C66—C69	122.1 (2)
C26—C25—C24	121.7 (3)	C65—C66—C69	120.3 (2)
C26—C25—H25	119.1	C62—C67—H67A	109.5
C25—C26—C30	121.1 (3)	C62—C67—H67B	109.5
C27—C26—C25	118.8 (2)	C62—C67—H67C	109.5
C27—C26—C30	120.2 (3)	H67A—C67—H67B	109.5
C26—C27—H27	119.1	H67A—C67—H67C	109.5
C26—C27—C28	121.8 (2)	H67B—C67—H67C	109.5
C28—C27—H27	119.1	C64—C68—H68A	109.5
C23—C28—C27	117.8 (2)	C64—C68—H68B	109.5
C23—C28—C31	122.1 (2)	C64—C68—H68C	109.5
C27—C28—C31	120.1 (2)	H68A—C68—H68B	109.5
C24—C29—H29A	109.5	H68A—C68—H68C	109.5
C24—C29—H29B	109.5	H68B—C68—H68C	109.5
C24—C29—H29C	109.5	C66—C69—H69A	109.5
H29A—C29—H29B	109.5	C66—C69—H69B	109.5
H29A—C29—H29C	109.5	C66—C69—H69C	109.5
H29B—C29—H29C	109.5	H69A—C69—H69B	109.5
C26—C30—H30A	109.5	H69A—C69—H69C	109.5
C26—C30—H30B	109.5	H69B—C69—H69C	109.5
C26—C30—H30C	109.5	N8—C70—H70A	109.3
H30A—C30—H30B	109.5	N8—C70—H70B	109.3
H30A—C30—H30C	109.5	N8—C70—C71	111.58 (18)
H30B—C30—H30C	109.5	H70A—C70—H70B	108.0
C28—C31—H31A	109.5	C71—C70—H70A	109.3
C28—C31—H31B	109.5	C71—C70—H70B	109.3
C28—C31—H31C	109.5	C72—C71—C70	119.7 (2)
H31A—C31—H31B	109.5	C76—C71—C70	121.4 (2)
H31A—C31—H31C	109.5	C76—C71—C72	118.9 (2)
H31B—C31—H31C	109.5	C71—C72—H72	119.8
N4—C32—H32A	109.0	C73—C72—C71	120.3 (3)
N4—C32—H32B	109.0	C73—C72—H72	119.8
N4—C32—C33	112.72 (18)	C72—C73—H73	119.9
H32A—C32—H32B	107.8	C74—C73—C72	120.2 (3)
C33—C32—H32A	109.0	C74—C73—H73	119.9
C33—C32—H32B	109.0	C73—C74—H74	120.2
C34—C33—C32	121.0 (2)	C73—C74—C75	119.7 (3)
C38—C33—C32	119.8 (2)	C75—C74—H74	120.2
C38—C33—C34	119.2 (2)	C74—C75—H75	119.8
C33—C34—H34	119.9	C76—C75—C74	120.4 (3)
C35—C34—C33	120.2 (2)	C76—C75—H75	119.8
C35—C34—H34	119.9	C71—C76—H76	119.7
C34—C35—H35	119.9	C75—C76—C71	120.6 (3)
C36—C35—C34	120.3 (2)	C75—C76—H76	119.7
C36—C35—H35	119.9	Cl3—C77—H77A	110.3
C35—C36—H36	120.1	Cl3—C77—H77B	110.3
C35—C36—C37	119.8 (2)	C77^i^—C77—Cl3	107.0 (12)
C37—C36—H36	120.1	C77^i^—C77—H77A	110.3
C36—C37—H37	120.1	C77^i^—C77—H77B	110.3
C36—C37—C38	119.9 (2)	H77A—C77—H77B	108.6
C38—C37—H37	120.1	Cl3B—C77B—H77C	109.1
C33—C38—C37	120.5 (2)	Cl3B—C77B—H77D	109.1
C33—C38—H38	119.7	C77B^i^—C77B—Cl3B	112.6 (9)
C37—C38—H38	119.7	C77B^i^—C77B—H77C	109.1
N5—C39—Ag2	127.91 (16)	C77B^i^—C77B—H77D	109.1
N6—C39—Ag2	128.31 (16)	H77C—C77B—H77D	107.8
N6—C39—N5	103.66 (19)		
			
N1—C2—C3—N2	0.2 (2)	C32—N4—C20—N3	177.14 (19)
N1—C13—C14—C15	−99.3 (3)	C32—N4—C21—C22	−177.4 (2)
N1—C13—C14—C19	82.5 (3)	C32—C33—C34—C35	176.4 (2)
N2—C4—C5—C6	177.6 (2)	C32—C33—C38—C37	−176.72 (19)
N2—C4—C5—C10	−1.2 (4)	C33—C34—C35—C36	0.3 (4)
N2—C4—C9—C8	−178.58 (19)	C34—C33—C38—C37	0.8 (3)
N2—C4—C9—C12	0.1 (3)	C34—C35—C36—C37	0.7 (4)
N3—C23—C24—C25	179.8 (2)	C35—C36—C37—C38	−0.9 (4)
N3—C23—C24—C29	0.7 (4)	C36—C37—C38—C33	0.2 (3)
N3—C23—C28—C27	−178.5 (2)	C38—C33—C34—C35	−1.0 (4)
N3—C23—C28—C31	1.1 (4)	C39—N5—C40—C41	−0.5 (3)
N4—C21—C22—N3	−0.1 (3)	C39—N5—C42—C43	98.1 (3)
N4—C32—C33—C34	76.8 (3)	C39—N5—C42—C47	−81.3 (3)
N4—C32—C33—C38	−105.8 (2)	C39—N6—C41—C40	0.2 (3)
N5—C40—C41—N6	0.2 (3)	C39—N6—C51—C52A	−72.1 (12)
N5—C42—C43—C44	−177.9 (2)	C39—N6—C51—C52B	−68.1 (10)
N5—C42—C43—C48	1.9 (3)	C40—N5—C39—Ag2	−175.70 (17)
N5—C42—C47—C46	178.0 (2)	C40—N5—C39—N6	0.6 (3)
N5—C42—C47—C50	−1.6 (3)	C40—N5—C42—C43	−87.4 (3)
N6—C51—C52A—C53A	147 (2)	C40—N5—C42—C47	93.2 (3)
N6—C51—C52A—C57A	−39 (3)	C41—N6—C39—Ag2	175.78 (17)
N6—C51—C52B—C53B	135.9 (14)	C41—N6—C39—N5	−0.5 (3)
N6—C51—C52B—C57B	−45 (2)	C41—N6—C51—C52A	109.2 (12)
N7—C59—C60—N8	0.0 (3)	C41—N6—C51—C52B	113.2 (10)
N7—C61—C62—C63	−176.8 (2)	C42—N5—C39—Ag2	−0.5 (3)
N7—C61—C62—C67	3.8 (4)	C42—N5—C39—N6	175.8 (2)
N7—C61—C66—C65	177.2 (2)	C42—N5—C40—C41	−175.5 (2)
N7—C61—C66—C69	−1.9 (4)	C42—C43—C44—C45	0.0 (4)
N8—C70—C71—C72	−98.4 (3)	C43—C42—C47—C46	−1.4 (3)
N8—C70—C71—C76	81.7 (3)	C43—C42—C47—C50	179.1 (2)
C1—N1—C2—C3	0.0 (3)	C43—C44—C45—C46	−1.4 (4)
C1—N1—C13—C14	−94.7 (3)	C43—C44—C45—C49	177.5 (2)
C1—N2—C3—C2	−0.4 (3)	C44—C45—C46—C47	1.5 (4)
C1—N2—C4—C5	96.2 (3)	C45—C46—C47—C42	−0.1 (4)
C1—N2—C4—C9	−85.1 (3)	C45—C46—C47—C50	179.4 (2)
C2—N1—C1—Ag1	172.57 (16)	C47—C42—C43—C44	1.5 (3)
C2—N1—C1—N2	−0.3 (2)	C47—C42—C43—C48	−178.7 (2)
C2—N1—C13—C14	86.6 (3)	C48—C43—C44—C45	−179.8 (2)
C3—N2—C1—Ag1	−172.80 (15)	C49—C45—C46—C47	−177.4 (2)
C3—N2—C1—N1	0.4 (2)	C51—N6—C39—Ag2	−3.1 (3)
C3—N2—C4—C5	−89.5 (3)	C51—N6—C39—N5	−179.4 (2)
C3—N2—C4—C9	89.2 (3)	C51—N6—C41—C40	179.1 (2)
C4—N2—C1—Ag1	2.1 (3)	C51—C52A—C53A—C54A	173.0 (18)
C4—N2—C1—N1	175.35 (18)	C51—C52A—C57A—C56A	−171 (2)
C4—N2—C3—C2	−175.3 (2)	C51—C52B—C53B—C54B	175.8 (13)
C4—C5—C6—C7	0.9 (4)	C51—C52B—C57B—C56B	−178.0 (17)
C5—C4—C9—C8	0.1 (3)	C52A—C53A—C54A—C55A	−2 (2)
C5—C4—C9—C12	178.8 (2)	C53A—C52A—C57A—C56A	3 (3)
C5—C6—C7—C8	0.3 (4)	C53A—C54A—C55A—C56A	1.9 (16)
C5—C6—C7—C11	−178.5 (2)	C54A—C55A—C56A—C57A	0.5 (17)
C6—C7—C8—C9	−1.3 (4)	C55A—C56A—C57A—C52A	−3 (2)
C7—C8—C9—C4	1.1 (3)	C57A—C52A—C53A—C54A	−1 (3)
C7—C8—C9—C12	−177.6 (2)	C52B—C53B—C54B—C55B	2.0 (16)
C9—C4—C5—C6	−1.0 (4)	C53B—C52B—C57B—C56B	1 (3)
C9—C4—C5—C10	−179.8 (2)	C53B—C54B—C55B—C56B	1.8 (12)
C10—C5—C6—C7	179.6 (3)	C54B—C55B—C56B—C57B	−4.1 (13)
C11—C7—C8—C9	177.4 (2)	C55B—C56B—C57B—C52B	2.6 (19)
C13—N1—C1—Ag1	−6.3 (3)	C57B—C52B—C53B—C54B	−3 (2)
C13—N1—C1—N2	−179.10 (18)	C58—N7—C59—C60	−0.3 (3)
C13—N1—C2—C3	178.86 (19)	C58—N7—C61—C62	73.9 (3)
C13—C14—C15—C16	−177.0 (3)	C58—N7—C61—C66	−105.0 (3)
C13—C14—C19—C18	176.4 (2)	C58—N8—C60—C59	0.4 (3)
C14—C15—C16—C17	0.4 (5)	C58—N8—C70—C71	−97.7 (3)
C15—C14—C19—C18	−1.8 (4)	C59—N7—C58—Ag2	−176.80 (17)
C15—C16—C17—C18	−1.5 (5)	C59—N7—C58—N8	0.5 (3)
C16—C17—C18—C19	0.9 (4)	C59—N7—C61—C62	−105.1 (3)
C17—C18—C19—C14	0.8 (4)	C59—N7—C61—C66	76.1 (3)
C19—C14—C15—C16	1.2 (4)	C60—N8—C58—Ag2	176.64 (17)
C20—N3—C22—C21	−0.4 (3)	C60—N8—C58—N7	−0.5 (3)
C20—N3—C23—C24	−105.6 (3)	C60—N8—C70—C71	78.2 (3)
C20—N3—C23—C28	74.5 (3)	C61—N7—C58—Ag2	4.1 (3)
C20—N4—C21—C22	0.6 (3)	C61—N7—C58—N8	−178.6 (2)
C20—N4—C32—C33	−99.9 (2)	C61—N7—C59—C60	178.8 (2)
C21—N4—C20—Ag1	170.81 (16)	C61—C62—C63—C64	0.1 (4)
C21—N4—C20—N3	−0.8 (2)	C62—C61—C66—C65	−1.6 (4)
C21—N4—C32—C33	77.8 (3)	C62—C61—C66—C69	179.3 (3)
C22—N3—C20—Ag1	−171.10 (16)	C62—C63—C64—C65	−2.5 (5)
C22—N3—C20—N4	0.7 (2)	C62—C63—C64—C68	176.6 (3)
C22—N3—C23—C24	77.7 (3)	C63—C64—C65—C66	2.9 (5)
C22—N3—C23—C28	−102.3 (3)	C64—C65—C66—C61	−0.9 (4)
C23—N3—C20—Ag1	11.7 (3)	C64—C65—C66—C69	178.2 (3)
C23—N3—C20—N4	−176.4 (2)	C66—C61—C62—C63	2.0 (4)
C23—N3—C22—C21	176.7 (2)	C66—C61—C62—C67	−177.4 (3)
C23—C24—C25—C26	−1.4 (4)	C67—C62—C63—C64	179.5 (3)
C24—C23—C28—C27	1.5 (4)	C68—C64—C65—C66	−176.2 (3)
C24—C23—C28—C31	−178.8 (3)	C70—N8—C58—Ag2	−6.9 (3)
C24—C25—C26—C27	1.6 (4)	C70—N8—C58—N7	175.89 (19)
C24—C25—C26—C30	−178.2 (3)	C70—N8—C60—C59	−176.1 (2)
C25—C26—C27—C28	−0.1 (4)	C70—C71—C72—C73	−180.0 (3)
C26—C27—C28—C23	−1.4 (4)	C70—C71—C76—C75	179.7 (2)
C26—C27—C28—C31	179.0 (3)	C71—C72—C73—C74	0.7 (5)
C28—C23—C24—C25	−0.2 (4)	C72—C71—C76—C75	−0.1 (4)
C28—C23—C24—C29	−179.4 (3)	C72—C73—C74—C75	−0.9 (5)
C29—C24—C25—C26	177.8 (3)	C73—C74—C75—C76	0.7 (5)
C30—C26—C27—C28	179.6 (3)	C74—C75—C76—C71	−0.1 (4)
C32—N4—C20—Ag1	−11.3 (3)	C76—C71—C72—C73	−0.1 (4)

Symmetry code: (i) −*x*, −*y*+1, −*z*.

### µ-Chlorido-bis{[1-benzyl-3-(2,4,6-trimethylphenyl)imidazol-2-ylideneκC]silver(I)} chloride 1,2-dichloroethane hemisolvate Hydrogen-bond geometry (Å, º)

**Table:**

*D*—H···*A*	*D*—H	H···*A*	*D*···*A*	*D*—H···*A*
C2—H2···Cl2	0.95	2.80	3.573 (2)	140 (1)
C8—H8···Cl2	0.95	2.81	3.669 (2)	157 (1)
C13—H13*A*···Cl2	0.99	2.91	3.784 (2)	147 (1)
C21—H21···Cl2	0.95	2.78	3.567 (2)	141 (1)
C38—H38···Cl2	0.95	2.90	3.779 (2)	155 (1)
C41—H41···Cl2	0.95	2.66	3.419 (2)	137 (1)
C70—H70*A*···Cl2	0.99	2.74	3.634 (2)	151 (1)
